# What makes a health system good? From cost-effectiveness analysis to ethical improvement in health systems

**DOI:** 10.1007/s11019-023-10149-9

**Published:** 2023-05-12

**Authors:** James Wilson

**Affiliations:** Department of Philosophy, University College London, Gower Street, WC1E 6BT London, UK

**Keywords:** Operations research, Cost-effectiveness analysis, Resource allocation, Complex systems, Scarcity, First-come first-served

## Abstract

Fair allocation of scarce healthcare resources has been much studied within philosophy and bioethics, but analysis has focused on a narrow range of cases. The Covid-19 pandemic provided significant new challenges, making powerfully visible the extent to which health systems can be fragile, and how scarcities within crucial elements of interlinked care pathways can lead to cascading failures. Health system resilience, while previously a key topic in global health, can now be seen to be a vital concern in high-income countries too. Unfortunately, mainstream philosophical approaches to the ethics of rationing and prioritisation provide little guidance for these new problems of scarcity. Indeed, the cascading failures were arguably exacerbated by earlier attempts to make health systems leaner and more efficient. This paper argues that health systems should move from simple and atomistic approaches to measuring effectiveness to approaches that are holistic both in focusing on performance at the level of the health system as a whole, and also in incorporating a wider range of ethical concerns in thinking about what makes a health system good.

## Introduction

To say that a resource is scarce is to say that there is not enough of it to achieve one or more desired end. Financial scarcity is omnipresent for policymakers; within health systems, other scarce resources include clinicians’ time, hospital beds, and medical equipment. When resources are scarce, policymakers do not just need to decide what to do, but also to determine fairly what not to do. As health interventions are often life-saving or at least game-changing for a patient’s quality of life, the need to decline funding for some health interventions puts pressure on policymakers to be able to give a reasoned explanation to those citizens denied access.^1^

COVID-19 placed unprecedented pressures on health systems. While fair allocation of scarce healthcare resources has been much studied within philosophy and bioethics, the nature of the scarcities during the pandemic provided significant new challenges. One reason was that some scarcities were of kinds that had not much been discussed before (for example, personal protective equipment, and ventilators). Other kinds of scarcities such as staff time became salient in new ways, as health systems struggled to meet significantly increased patient need with a depleted clinician workforce.

This paper focuses on a fundamental challenge that the pandemic made painfully visible, but for which existing philosophical approaches offer little guidance: namely, the extent to which scarcities within crucial elements of interlinked health systems can lead to cascading failures that cause widespread harm and loss of life. Health system resilience, while previously a key topic in global health, can now be seen to be a vital concern in high-income countries too.^2^

I use the concept of *health system improvement* to refer to the processes by which policymakers aim to deliver the best health system possible, given financial, broader resource, and other constraints. I construe improvement in a deliberately broad and inclusive way, with the intention that it will be uncontentious (indeed truistic) that policymakers should aim to improve health systems. What will be contentious is the principles that should guide improvement, and what improvement will require in practice. Thus, while the concept of health system improvement serves to indicate neutrally a topic of enquiry, there will be a variety of competing conceptions of health system improvement.^3^

Health systems are above all *systems*, and all systems transform inputs into outputs. The inputs may be of various kinds. In the context of healthcare, key inputs include money, staff time, pharmaceuticals, and medical equipment. In non-healthcare contexts, raw materials such as steel or bauxite, or schemas such as a blueprint for a building or a mobile phone may be salient inputs. The outputs of health systems are similarly varied—from a successfully excised appendix, to a new medical device, to a reduction in ethnic disparities in healthcare outcomes. The outputs of one system or set of processes will serve as the inputs for others, and this occurs iteratively. Thus, the published results of a randomised clinical trial are outputs of a process of research, but also serve as inputs to a systematic review of the literature. The systematic review may then serve as an input to a process by which best-practice guidelines are revised.

I use the idea of a *flow* in a broad sense, to refer to a process by which inputs are transformed into outputs within a system. In the context of a healthcare system, prominent flows include those of money to reimburse physicians or hospitals for providing care, referral journeys as patients shift from one health provider to another in pursuit of a diagnosis and care, and the processes by which health information gathered from care of individual patients is curated and repurposed for planning and research. These flows are only a fraction of those that could be isolated and studied. Understanding how different flows interconnect within a health system is itself a major undertaking, and attempts to do so will be constrained by data availability and data quality.^4^

Few people care about the inner workings of their washing machine—so long as it cleans and spins their clothes. So, where a system is functioning in a way that meets or exceeds expectations, then it might seem sufficient to treat it as a “black box”, which takes inputs and produces outputs, without any further need to understand the flows by which such transformations occur. There are obvious reasons for avoiding such complacency in the delivery of public services: further scrutiny may determine significant inefficiencies, or structural inequalities, with the implication that the expectations of functioning placed on the system were too low. In any case, the constant presence of scarcity within health systems naturally leads to questions about the structure of flows, especially by those who are denied access to care that would be beneficial for them. And it is not just scarcity that leads to concerted calls for better understanding of the different flows by which inputs are converted into outputs. For example, where despite best endeavours, cancer survival rates in a health system show significant disparities on the basis of ethnicity, this may prompt detailed scrutiny of the systemic factors responsible, including the social determinants of health, the care journeys of different groups of patients, and how treatment pathways could be improved.

What is the best conception of improvement for a health system? Some ethical theories might naturally suggest generic answers that can be worked out in the abstract and then applied both to health systems and a wide variety of other systems—for example, that a system should seek to maximise wellbeing, or that it should seek to meet the greatest sum of strength-weighted relevant claims (Voorhoeve 2014).

This paper argues that the best conception of improvement for a health system needs to be specific to the kind of endeavour that a healthcare system is. In order to explain why improvement of health systems requires bespoke contextual ethical analysis, it is important to distinguish *means-improvement*, and *values-improvement*. Means-improvement involves mapping the ways in which inputs are converted into outputs via different flows, and examining whether these could be reconfigured to allow the system as a whole to better to achieve the values it aims to instantiate and promote. There will be a myriad of different ways of arranging these different flows. Some of these will be faster, or require less resources, or involve fewer mistakes, or will be more resilient than others.

Values-improvement involves specifying and reconciling the values that a system should instantiate and promote. The precise formulation of the values of a health system are part of what needs to be determined in articulating the best conception of improvement, but it is a substantive, and questionable, assumption to make that all these values should be generic rather than health-system specific. The deep cultural significance of birth, suffering, and death have long shaped medicine as a social practice, for example through articulations of the nature and importance of care, the role-specific obligations of clinicians, and duties of medical confidentiality. I will have more to say about these points in Sect. 6, but for now, the important point to make is that healthcare is taken by policymakers, clinicians and citizens more broadly to entail a set of *sui generis* ethical considerations. It would be odd to assume that health system improvement should ignore this fact. Taking it seriously may require us to think of means-improvement and values-improvement in healthcare as entwined.

Improving a system as complex as a health system requires mapping systemic interactions, and deciding how to prioritise and to combine a range of goods, which for contextual reasons may be in partial conflict, and which may also be specific to healthcare. Given the sheer number of flows, and the complexity and density of the interlinkages of these flows, it would be unrealistic to suppose that the end result of a process of improvement would be a health system perfect for all flows and all purposes. Even a very detailed flow map will be significantly more coarse grained than the world that we are attempting to explain and understand. Even leaving this epistemic problem on one side, it is implausible to think that there could be a single cardinal number that would adequately sum up how well a health system is performing, or which could be maximised subject to constraints (Wilson 2017, 2021).

These points become obvious when we examine just a few of the tradeoffs that all health systems must make in specifying and pursuing their goals. Providing a health system that can cope with spikes in demand without degradation in quality of service is important, but so is ensuring that public resources invested provide good value for money. Focusing only on value for money encourages policymakers to focus on eliminating waste and spare capacity, but a health system that is designed according to just-in-time principles, and hence has little spare capacity across each element of its supply chain, is likely to find it difficult to flex to meet the demands of a public health emergency. A system that is maximally cost-effective is also typically one that is brittle. (Vardi 2022) All health systems need to ensure that citizens are confident that their health information will be kept confidential; but all modern health systems also need to be able to use health data to deliver care to their patients, and in identifiable or anonymised or aggregate forms for planning and research purposes. Ensuring that patient confidentiality can be maintained while allowing data to flow without too much encumbrance for necessary planning and research is not a straightforward task, and how to balance the considerations in play is likely to be a topic of reasonable disagreement, both within the same health system and between health systems (Taylor and Wilson 2019).

There also are values that are widely believed contribute towards a high-quality health system, but which are not valued merely for their health effects (Wilson 2017). For example, it is reasonable to think that patients should be able to receive surgery in a hospital close enough for them to be easily visited by family and friends. This value of proximity and convenience will sometimes be in tension with the equally reasonable goal of minimising rates of surgical complications: surgeons who perform a given procedure twice a week will tend to have fewer complications than those who perform it twice a year, and this pushes policymakers towards greater centralisation of complex surgeries. It is not obvious how to resolve this tension between convenience and best surgical outcomes, but the fact that doing so is not straightforward strongly suggests that our account of a high-quality health system should be broader than one that produces good health outcomes. How to theorise quality within healthcare is itself a topic of significant importance (Cribb et al. 2020).

The rest of this article argues for a shift in philosophical thinking about health system improvement from conceptions that foreground cost-effectiveness analysis, to a more flexible, iterative, and contextual conception of improvement in which means-improvement and values-improvement are seen as intertwined. Cost-effectiveness analysis aims to allow policymakers both to quantify the size of health benefits that can be created through different interventions, and to compare interventions in terms of a common measure. As it seemingly provides a rigorous way of measuring and comparing the effects of health interventions, it is widely assumed that cost-effectiveness should play a key role within means-improvement in health systems. However, the role that cost-effectiveness ought to play in health system improvement depends both on how effective it is for means-improvement, and the extent to which cost-effectiveness is congruent with the system’s values as articulated via a process of values-improvement.

Section 2 argues that using cost-effectiveness analysis as the only value in means-improvement in a health system (what I call a comprehensive approach to cost-effectiveness), requires making four assumptions, which I label *measurability*, *single synoptic decision procedure*, *external validity*, and *static ranking*. The position that arises from combining all four claims is that health can be measured on a cardinal scale (*measurability*), which allows numerical comparisons to be made across all health states; such measurements allow for a single correct decision procedure for health prioritisation (*single synoptic decision procedure*); measurement of cost-effectiveness made in one context can be transferred without difficulty to other contexts (*external validity*), and that rankings produced by prioritisation processes are static rather than dynamically changing (*static ranking*).

Sections 3 and 4 look in more detail at the relationship between the use of cost-effectiveness analysis, and a health system’s ability to match available healthcare resources to eligible healthcare need, using the English National Health Service (NHS) as a case study. The NHS is well-known for the explicitness of the prioritisation procedures through which new interventions are introduced into the health system, and the role that cost-effectiveness analysis plays within these. Section 3 argues that while the NHS places a heavier emphasis on cost-effectiveness analysis than most other health systems, it nonetheless runs a mixed economy in which only a minority of decisions is shaped by formal measurement of cost-effectiveness. This mixed approach has not prevented a significant increase in waiting times and other forms of implicit rationing, which have led to widespread harm; these failures are not incidental, but follow from the approach adopted. This invites the question whether the problem has been not enough cost-effectiveness analysis, or whether what is required is an approach to improvement that looks beyond cost-effectiveness.

Section 4 argues that significantly increasing the range and reach of cost-effectiveness analysis would provide a good approach to means-improvement only if *external validity* and *static ranking* held, and doing so allowed the system as a whole to move in the direction of its values, as articulated and reconciled through processes of values-improvement. By looking at a case study on cataract waiting lists, and one on cascading harms caused by ambulance waiting times, I argue that neither *external validity* nor *static ranking* is correct within large-scale and interconnected health systems. We need a more complex model of fairness in resource allocation, which focuses on a range of ethical values beyond cost-effectiveness, and which is responsive to the fact that the effectiveness of interventions is often holistically determined.

Sections 5 and 6 sketch an account of health system improvement which rethinks health prioritisation beyond the four assumptions. Section 5 argues that means-improvement and values-improvement are often entangled and reciprocal, rather than discrete and hierarchical. It follows that improvement should be thought of as continual and iterative, rather than something done at a particular time or place and then applied. Section 6 explores the process of values-improvement in healthcare, arguing that improving a health system is different from improving a factory, or a football team. The values inherent in healthcare as a social practice, and the role of democratic deliberation in setting the goals for healthcare institutions help to explain why. Section 7 concludes.

## Four assumptions of cost-effectiveness based improvement

Using cost-effectiveness analysis as the sole factor in health system improvement would require policymakers to make four assumptions about health, how to reconcile competing ethical values, and the reliability of cost-effectiveness analysis across different contexts and times. The first assumption is *measurability*, namely that whatever makes healthcare valuable can be measured on a cardinal scale using a validated measure, and that numerical comparisons can be made across all such states. Without a common denominator in terms of which the effects of different interventions can be reliably measured, policymakers would not be able to determine which of two interventions for different diseases would create the greatest health benefit for a given sum of money.

Despite the ubiquity of the assumption of *measurability*, it is neither obvious that health and illness are cardinally measurable, nor there is any alternative specification of the aim of health policy that is cardinally measurable either. For example, influenza, depression, spina bifida, herpes, and rheumatoid arthritis are all ways of being ill, but they each affect us in very different ways. It is a substantive assumption to make that there is (or can be assumed to be without losing anything ethically significant) one common thing, “health” that is reduced in each of these different cases, and which can be measured on a single scale using a measure such as the Quality-Adjusted Life Year (QALY) or Disability-Adjusted Life Year (DALY). Owing to these kinds of concerns, Hausman (2015) argues that it would be more accurate to think of measures such as the QALY or DALY as measures of the *value* that populations place on different health states, rather than measures of which state contains more health.

Regardless of whether we take measures such as the QALY or DALY to be measuring health, or the value of health, it is much less plausible to think that there is a single and uniquely correct way of measuring health or wellbeing, than a plurality of different valid ways of so doing (Mitchell and Alexandrova 2021). For example, the QALY is a well worked out measure of health related quality of life, but examining the methodology by which the QALY is operationalised makes clear that value judgements and assumptions which could be otherwise are made at several points. These include the idea that all health states can be classified along five dimensions, that health states (even from very different diseases or disabilities) which are classified the same on these five dimensions should be treated the same for the purpose of resource allocation, and that the values used to score how bad it is to be in one of these health states should be determined by members of the public who have probably not been in such a health state themselves (Pettitt et al. 2016). This is not to claim that any of these decisions is indefensible, but that in each case there are reasonable alternatives to the decisions that were taken, and each of these reasonable alternatives might lead to different answers to the question of how cost-effective a particular intervention is, and how its cost-effectiveness compares to other potential interventions. The DALY is constructed and validated in a very different way from the QALY, but also makes a number of evaluative assumptions that could reasonably be questioned (Solberg et al. 2020). Similar concerns would apply to all other measures of health.

The second assumption is that information about different health states as they affect different persons can all be combined in a single decision procedure, which can then be used to determine priorities in an uncontroversially fair way. Call this *single synoptic decision procedure*. Such a procedure could be maximisation of expected QALYs, or it could be a more complex one involving, for example equity weighted maximisation (Cookson et al. 2017). *Single synoptic decision procedure* is widely agreed to be controversial. For example, it is unclear whether one unit (e.g. one QALY) should count the same regardless of the disease it arises from, how badly off the patients are who are affected, and how significant the benefit is to each individual who would receive it. Moreover, there is a range of other values that are often thought to be relevant to health systems improvement, such as inclusion, and environmental sustainability that are only indirectly connected to health achievement. Prominent scholars, including Norman Daniels, argue that it is not possible to resolve a range of underlying ethical disagreements about tradeoffs in resource allocation through philosophical reasoning alone (Daniels 1994). If they are correct, a turn to procedural reason is required, which may provide answers that are *reasonable*, but which cannot provide answers that can be claimed to be uniquely correct (Daniels and Sabin 1997; Rumbold et al. 2016).

The third assumption is *external validity*, namely that assessments of cost-effectiveness can be performed in one context and then transferred without significant difficulty to the real-world context of the policymaker’s health system. The accuracy of measurements of *cost*-effectiveness depends on the accuracy of measurements of effectiveness. Accuracy of measurements of effectiveness across contexts depends on either the underlying sameness of causal effects across these contexts, or at least being able accurately to infer how effectiveness will differ in the policymaker’s context when compared to the experimental context. As has been widely discussed in the context of randomised controlled trials, there may be a significant gap between the effects of an intervention in an experimental context, and the effects that the same intervention will have in a real-world context (Deaton and Cartwright 2018). Transferability of results from one context to another is a widely recognised challenge in healthcare research, but it often does not receive sufficient attention within cost-effectiveness analysis. In particular, cost-effectiveness analysis in healthcare often assumes that cost-effectiveness of interventions that are given as part of an overall plan of care can be calculated in the abstract, and that once calculated this can be treated as a reliable measure that can be plugged in unchanged to decisions in a wide variety of healthcare contexts.

The fourth assumption is *static ranking*, namely that rankings in a prioritisation process—regardless of whether these rankings are of the strength of claims to receive a particular expensive treatment, or of how much resource should be devoted to a range of services such as those involving cardiovascular disease—will not change unexpectedly over the period governed by the resource allocation. Change in the variables that are relevant to prioritisation, and the options between which decisions must be taken, either do not occur or such changes are known and can be factored into the prioritisation process. Those who rely on *static ranking* do not deny that significant changes that are relevant to prioritisation of healthcare resources do occur—whether through innovation (game-changing new drugs), novel pathogens, or changing demographics, but they tend to treat these possibilities as external to processes of prioritisation. Their assumption is that prioritisation can be theorised as working on atemporal snapshots without losing anything of ethical significance; or as Arthur (2021) explains in a helpful recent article, economics can be done with nouns only, rather than verbs.

We have seen that that *measurability* and *single synoptic decision procedure* are already widely acknowledged to be controversial. Conceptions of health system improvement that make use of cost-effectiveness should acknowledge this. In so doing, they can adopt a view of cost-effectiveness analysis that sees it as a pragmatically useful, but imperfect tool, rather than the only ethically defensible approach to health system improvement. Of course, doing so places renewed emphasis on questions of values-improvement, and the extent to which a heavy emphasis on means-improvement via cost-effectiveness analysis is compatible with a health system’s values. For example, if cost-effectiveness is used as the basis for a prioritisation policy that would not fund some life-saving though very expensive drug, and it is admitted that this prioritisation process is *a* reasonable process, but only one of a range of reasonable processes by which the challenge of fair health prioritisation could be met, then a second-order challenge of fairness can be raised. Where prioritisation processes lead to individuals being denied access to life-saving care that they would have received on a different but equally reasonable approach to resource allocation, such individuals are likely to question the ethical justifiability of the processes that have been adopted. And to the extent that the answer to such questions is to acknowledge that other approaches that would lead to different rankings are equally as reasonable, it is not clear that this will provide an ethically satisfying answer for those denied access to life-saving care.

*External validity* and *static ranking* are also controversial, but the significance of this point is much less frequently discussed within the literature on healthcare prioritisation. It is a substantive, and usually false, assumption that the effects (and hence cost-effectiveness) of an intervention are *atomic*, i.e. that they are the same everywhere the intervention is undertaken, rather than shifting in response to changes elsewhere in health systems. For example, the effectiveness of drugs for chronic diseases is obviously impaired if patients do not adhere to the recommended drug schedule. However, patients’ ability to adhere is fragile and contextual (Stutzin Donoso 2021), with the figure regularly being reported that only around 50% of chronic disease patients correctly adhere to treatment. The effectiveness (and cost-effectiveness) of a drug in practice depends on, among other things, on the level of support that patients have in adhering to treatment. These points about context and support mechanisms apply widely to measures of real-world effectiveness.

Similarly, it is common, rather than rare, for circumstances to change in ways that require rankings delivered in prioritisation processes to be reconfigured within rounds of resource allocation, rather than only at the beginning of such a period. The way that services are prioritised within a hospital will presuppose (even if implicitly) that the flow of patients will fall within certain parameters—for example that 300 beds will be sufficient for the hospital as a whole even at moments of high demand, and that the Accident and Emergency department will not regularly have to process more than 40 patients an hour. If the system needs to start operating outside of these parameters, then this may make a significant difference to what calibration of services will be overall most cost-effective. Where prioritisation itself involves waiting (as in a transplantation waiting list), the severity of patients’ conditions will alter over time, both relative to their own baseline health state when they entered the waiting list, and relative to other patients. A prioritisation ordering that *was* fair may not remain fair as patients’ conditions change.

Calling *external validity* and *static ranking* into question creates a deeper challenge for conceptions of improvement which rely heavily on cost-effectiveness than does questioning *measurability* and *synoptic decision procedure*. As will be examined in Sect. 4 onwards, if it is admitted that it is questionable to assume that cost-effectiveness analyses have external validity, and it is also admitted that prioritisation rankings will often be dynamic rather than static, then the obvious inference to draw would be that it is also questionable to assume that the cost-effectiveness of particular interventions can be tested centrally once and for all, and that doing so will make possible fair decisions about when and how such interventions should be made available throughout the health system. Rather, where cost-effectiveness analysis is used, it should be in a constrained and judicious way.

## Cost-effectiveness in theory and practice

Regardless of how a health system is financed, it is unlikely that it will be affordable for it to provide all the health related services that could be potentially beneficial to everyone, without anyone needing to pay out-of-pocket. One helpful way of conceptualising the choices to be made is via the World Health Organization’s universal health coverage (UHC) cube, which asks policymakers to consider three dimensions on which health systems can extend outwards as they move towards universal health coverage:

1. Include more groups within the health coverage, or extend eligibility among these groups.
2. Reduce cost-sharing and fees.
3. Include more services within the plan.

The UHC cube is most often used in the context of expanding the scope of covered care, where the question is how to expand the dimensions of the cube fairly (World Health Organization 2014). However, the model is also relevant for considering what to do where the entitlements to healthcare that are created by a healthcare package cannot in fact be met with the resources currently allocated. In a single-payer health system, policymakers need to find an equilibrium between the sum that citizens considered collectively are willing to pay through taxation, and the extent of the health coverage that citizens collectively want to receive. Insurance-based systems, to the extent that they involve solidarity-based risk pooling, face similar challenges (Voorhoeve 2018).

The distinction between explicit and implicit prioritisation helps us to illuminate the choices that must be made where there is a mismatch between the scale of entitlement to receive care, and the resources available to meet this demand. Explicit prioritisation requires policymakers to resolve scarcities by using transparent principles. This requires policymakers to confront a number of uncomfortable questions: if there is not enough resource to meet everyone’s healthcare needs, then the obvious implication is that some individuals will not be able to access treatments (potentially life-saving treatments) that they require. The difficulty and the contentiousness of these decisions is one reason why the National Institute for Health and Care Excellence (NICE) was set up in the UK (Timmins et al. 2016).

If demand for services systematically outstrips the system’s capacity to meet this demand, then an explicit approach to prioritisation will require either that the available pooled funds are increased, or that the scope of coverage is contracted on one or more of the dimensions of the UHC cube, or that flows are redesigned via means-improvement to increase throughput. Implicit rationing occurs when how to resolve problems of scarcity is not addressed systematically, or where excess demand is managed by measures that are not transparent. In many cases, implicit rationing can occur without explicit decision or endorsement: queuing and congestion are often simply the unwanted net effect of excess demand within parts of the system.

We can distinguish between *comprehensive* and *partial* approaches to cost-effectiveness analysis. In a comprehensive approach, *all* interventions are rated for cost-effectiveness, and these procedures can then be ranked using a synoptic decision procedure. Culyer (2016) provides a simple model, on the assumption that the relevant decision procedure is QALY maximisation:

1. Work out how cost-effective each intervention is in £ per QALY.
2. Order all the interventions in order of cost-effectiveness. (Culyer asks us to visualise this as ordering books from left-to-right on a bookshelf in order of height; where height would represent cost-effectiveness).
3. In funding interventions, start by funding the most cost-effective, and keep moving to the right of the shelf until the money runs out.
4. If the budget limit changes, the cost-effectiveness threshold for which interventions can be afforded also changes—rising as the budget increases, and falling as the budget decreases.

In a partial approach, the cost-effectiveness of some but not all interventions and processes is investigated, meaning that it is possible to rank only some, but not all, interventions and processes in terms of cost-effectiveness. Partial cost-effectiveness analysis does allow policymakers to compare the cost-effectiveness of those interventions that have been assessed. Such analyses will often be important and compelling—for example it might tell us that a generic statin is almost as clinically effective as a much more expensive branded and patented one, but a fraction of the price, and thus much more cost-effective. However, there is a range of decisions for which a partial approach to cost-effectiveness analysis provides limited help. For example, if there is a requirement to reduce the healthcare budget by 10%, but only a fraction of the existing interventions have been evaluated in cost-effectiveness terms, then policymakers will lack evidence on whether the cuts should fall equally on those interventions and processes that have been assessed and found to be of low cost-effectiveness, as on those that have not been evaluated.

Cost-effectiveness analysis plays a much more significant role within the NHS than in most other health systems, and NICE’s methods for health technology appraisal are influential and widely admired. Nonetheless, the model used within the NHS is a long way from Culyer’s bookshelf. First, the NHS’s model does not assume a single synoptic decision procedure: the appraisal process involves a range of additional values such as uncertainty, budget impact, and rarity, which interact with NICE’s assessment of incremental cost-effectiveness (Charlton 2022). Moreover, to the extent that NICE is focused on incremental cost-effectiveness, it operates a satisficing model of cost-effectiveness rather than a maximising one (Rumbold et al. 2016). That is to say, NICE aims to ensure that treatments should meet at least a minimum standard of cost-effectiveness before they are deployed widely in the NHS, not that the intervention will maximise cost-effectiveness. In addition, NICE is asked to examine the cost-effectiveness of some (but not all) interventions and drugs that are introduced into the NHS. There are many existing interventions and institutional structures for which no formal cost-effectiveness analysis has been done.

Thus, while it is often assumed that the NHS is a system in which explicit rationing on the basis of cost-effectiveness analysis plays a very large role, the reality is more complex. The satisficing model ensures that (unless there are special features that justify paying more), a new intervention should not usually cost more than £30,000 per QALY. However, the best available evidence suggests that on average it costs significantly less than £30,000 to create a QALY within the NHS. In a widely cited study, Claxton et al. (2015) calculated that the most likely value for the cost it takes to create one QALY within the NHS is £12,936, with an 89% probability that the figure is less than £20,000. The uncomfortable implication is that, even though policymakers do not have a comprehensive understanding of the cost-effectiveness of all interventions, many of the drugs approved by NICE would be expected to lead to losses of health benefits that are larger than those they create. That is to say, if the cost-effectiveness calculations are correct, introducing new interventions into the NHS often fails to improve cost-effectiveness, and leads to a net loss of QALYs relative to the status quo, even though it may have some other virtues, such as providing access to a range of more expensive treatments that will provide genuine benefits to patients.

We have seen that the NHS as it currently exists is a system with significant scarcity, which has as its entry point for new interventions a standard that is markedly less cost-effective than the average of interventions already performed. In addition, there is no systematic attempt to identify existing interventions that are being performed, but which are of low cost-effectiveness. The system as a whole has no very clear mechanisms for stopping doing interventions that are not cost-effective.

If the explicit prioritisation mechanisms that a health system introduces to adjust the size of its UHC cube to the available funding do not, in fact, succeed in matching capacity to demand, then implicit rationing will occur notwithstanding the fact that there is also some explicit prioritisation. A plausible working hypothesis is that allowing greater demand into the system as a whole than the system is able to service will lead to system congestion: ever increasing waiting times, both for scheduled surgery, and for other operational flows such as moving patients out of ambulances into hospital, or a service whose quality is degraded in other ways. It may also lead to other sequelae such as significant increases in unmet healthcare needs, and patients who can afford to pay for access to private treatment doing so. It is irresponsible for policymakers to allow a systemic gap to appear between the demands that individuals can make under the system, and the system’s ability to meet these demands. To the extent that partial cost-effectiveness analysis leads to such problems in practice, it can and should be ethically criticized.

This analysis does not yet show that there is anything undesirable about comprehensive cost-effectiveness analysis. It would be possible to argue that what it shows is that the NHS should avoid the ethical costs associated with implicit rationing by going further and deeper with explicit prioritisation, with the aim of moving to a comprehensive approach to cost-effectiveness analysis. This would be a good answer to how best to improve health systems only if it can safely be assumed that *external validity* and *static ranking* hold—to recall, that cost-effectiveness analyses have external validity, and health systems are not so dynamic as to involve frequent shifts in rankings produced by prioritisation procedures. By looking in more detail at some recent challenges that have occurred in the NHS, I shall suggest that neither assumption is safe to make. We should adopt a conception of health system improvement that is better attuned to the dynamic complexity of health systems, and which draws on a wider range of values to help improve system responses.

## From cost-effectiveness to system level functioning

As of the beginning of 2023, the NHS was facing worse waiting lists for scheduled operations than at any point in its history, and also struggling to meet targets on a range of indicators such as ambulance response times, and Accident and Emergency department waiting times.^5^ Waiting in line is often used, de facto, as a way of managing scarcity in low-stakes cases such as queuing at the supermarket (John and Millum 2020). However, there is no reason to think that healthcare needs which present earlier will per se be more urgent, or in other ways create a stronger claim (Wilson 2012). Queuing and waiting lists are thus not an effective way of allocating care on the basis of need. For example, it would be a very odd approach to take to running an Emergency Department to see patients strictly in order of arrival, thus requiring a patient rushed in with a stroke to wait behind individuals with minor scrapes and injuries who happened to arrive first.

A policy of first come, first served, prioritises not only *by time*, but *in time*. Patients’ health problems occur in time, and change over time. Some health problems will be self-limiting, as in the case of a cold, others will get worse, and still others remain constant. A fair allocation system will need to keep track of the effects on individuals’ need and severity of condition while they are waiting. Where demand systematically outstrips the ability to meet demand, and first come, first served, is used as a method of allocating resources, then waiting times will grow ever longer. If demand systematically outstrips ability to meet it, and those waiting become significantly worse (and more difficult to treat) while they are waiting, then first-come first-served is ethically inferior to a system of explicit prioritisation based on criteria that allows capacity to be matched to eligible need. Where first-come, first-served is used to manage access to treatments for progressive conditions, and demand systematically outstrips ability to meet it, then no patients will reach the front of the queue until their condition has already deteriorated significantly and this avoidable loss of health will grow as the queue lengthens.

Cataract surgery, in part because it is such a common and routine non-urgent operation, provides a useful example. The NHS target for all non-urgent operations is 18 weeks. These targets were struggling to be met pre-pandemic (in part because of the way in which urgent cases displace non-urgent ones in the system’s capacity), but since the pandemic, waiting times have got significantly worse. One survey, based on 12 hospital trusts, found that waiting times for cataract surgery had increased by 35% in 2019–20, and another 36% in 2020–21, with the result that the mean wait for cataract surgery was 278 days (nearly 40 weeks) in 2021 (Eyewire 2021). This is a mean, and so in some hospital trusts, waiting time is significantly longer than this.

Cataract is a progressive condition. The longer it is left before an operation, the greater the complexity of the operation and the greater likelihood of complications. Patients who wait longer for cataract surgery will suffer diminished quality of life in the meantime, and may for example lose the ability to drive, and be more prone to accidents from diminished visual acuity. More people will be diagnosed with cataracts each week. Waiting lists get longer to the extent that the rate at which people are taken off the waiting list (by receiving the operation, paying to have the operation privately, or dying, or emigrating) is slower than the rate at which patients are added to the waiting list.

Cases become more time-consuming to treat (and more prone to complications) as cataracts worsen, and cataracts worsen as patients wait longer for treatment. It is much more difficult to reduce a backlog than it is to maintain a system that is functioning well with short waiting times. A level of capacity that was enough to keep waiting lists static at a waiting time of 18 weeks would not be sufficient to keep waiting lists static at a waiting time of 9 months; waiting lists would continue to get longer, and patients’ eye health would be worse by the time they reached the front of the queue.

In short, a cataract operation service that makes people wait until their condition gets worse before they are treated will not only be less cost-effective, but less ethically defensible, than one that operates on patients earlier in their disease pathway. This seems to provide a strong reason to provide a better match between eligible demand, and ability to meet demand, than the NHS has recently been able to.^6^

Cataract operations are just one indicative treatment pathway within a health system. Problems such as cataract surgery waiting times not only occur across the NHS, but interact with and exacerbate each other. A modern healthcare system, if it is to work for patients, needs to be tightly interlinked. Integrated health and care systems require the ability to transfer patients fluidly across departmental, institutional and geographical boundaries (Meek 2018). Any blockage to so doing may lead to cascading failures and shortages elsewhere in the system. Thus, scarcity in one narrow domain can worsen scarcity elsewhere, for example by creating bottlenecks that worsen the performance of other parts of the system.

This is a problem of scarcity, but it’s not a problem of scarcity that can be solved easily, or very satisfactorily, by a greater emphasis on cost-effectiveness analysis, or even by adopting comprehensive cost-effectiveness analysis in the mode that Culyer (2016) recommends. We commit a fallacy of composition if we assume that the cost-effectiveness in practice of a complex system as a whole is a simple function of the cost-effectiveness of its component elements. Of course, it is markedly easier to measure the cost-effectiveness of each element of a tightly integrated system separately and in the abstract, than to measure each element’s contextual contribution to the working of a tightly integrated real-world system, but what is gained in ease of analysis will often be lost in accuracy.

Integrated care systems, let alone a national system like the English NHS as a whole, by their nature involve multiple interlinked subsystems, which interact both hierarchically and horizontally. Each of these elements at the different hierarchical levels involve multiple inputs, and each of these elements will usually interact with other elements above and below it in the hierarchy. Thus, a large hospital may be organised into wards, departments, and sites, with the staff at each site organised into hierarchically structured teams. Much work in healthcare involves working across or between hierarchical structures—for example, assembling a multidisciplinary team to plan care in a complex case, or discharging a patient from a hospital into a residential care facility managed by a different institution. And so, each element also interacts with a variety of other elements outside of its formal hierarchical structure. The outputs of each of these elements often serve as inputs for other elements of the health system—leading overall to a system that is densely interconnected by both hierarchical and non-hierarchical interactions. In short, the picture is one of a large number of interlinked subsystems, some of which are arranged hierarchically, and others that interact without formally specified chains of authority.

How effective the health system as a whole is at fulfilling core functions does not just depend on the performance of a particular element within the overall system, but on interactions of numerous elements, which are each linked with one another via multiple feedback loops. Mapping system-level capacity and understanding how patients, diagnoses and resources flow around the system and where the pinch points are, and how system resilience can be improved is vital. Doing so can require a significant amount of contextual knowledge and modelling. For example, the demand, capacity and vulnerability of the health system in Greater Manchester will be significantly different in detail to that in London. Nonetheless, there is enough commonality to pick out some systemic dependencies and bottlenecks which are widely reported across the NHS, and will no doubt also have some implications for other health systems.

Patients cannot be discharged from hospital unless there are sufficient resources for them to be cared for safely outside of hospital. However, lack of available resources in social care frequently leads to delays in patients getting discharged from hospitals. If patients who are well enough to be discharged, but cannot be discharged due to lack of social care resources, take up a significant proportion of hospital beds, then this makes it more difficult to move patients out of Accident and Emergency Departments onto wards. If there are delays in patients being moved out of Accident and Emergency departments, then this also impedes the ability to bring patients *into* Accident and Emergency Departments. Patients need to wait in ambulances outside hospitals before they can be admitted, which in turn takes ambulances out of service while they wait with patients in hospital car parks.^7^ This leads to worse response times for ambulances, which then leads to additional load on emergency call handlers, as they respond to repeated calls about ambulances that have not arrived.^8^ This is to take just one trajectory out of the myriad ways in which scarcities may ramify and exacerbate one another within a tightly integrated system with little spare capacity.

Obviously, no policymakers intended for all these patients to come to harm as a result of system congestion. The fact that harm is occurring on a large scale due to cascading scarcities within a system that makes use significant use of cost-effectiveness analysis raises difficult questions at a practical level. When does cost-effectiveness analysis help, and when does it hinder in improving health systems? At a theoretical level, the analysis gives some useful insights into things that any good health system would want to avoid, and provides strong reasons for thinking that successful means-improvement of health systems requires mapping of the different flows, resource dependencies and inter-relations at a system level. Nonetheless, acknowledging this still leaves the value choices that must be made in improving a health system significantly underdetermined. The next two sections aim to remedy this deficit.

## Means-improvement and values-improvement are entangled

Much of the discussion so far has been about opening up, rather than closing down, discussions of health system improvement. I have made the case that centring improvement attempts on improving cost-effectiveness is a sensible strategy only if *measurabillty, single synoptic decision procedure*, *external validity*, and *static ranking* all hold. We have seen that each of these assumptions prove to be implausible in a range of real-world contexts. Nonetheless, comprehensive cost-effectiveness analysis at least has the advantage that it offers *a* way of making otherwise very difficult problems more tractable by introducing a common measure, and using this measure as the basis of a prioritisation process. So it is easy to see how, absent an obviously better alternative, it might continue to exert an inexorable pull on the imaginations of policymakers.

This section and the next sketch an alternative conception of improvement that does not require any of the four assumptions, and which is normatively rigorous, democratically informed, and practicable. The aim is not to advocate for the abandonment of tools such as cost-effectiveness analysis, but to reframe them as one among a plurality of tools by which health system performance can be measured and means-improved. This section clarifies the relationship between means-improvement and values-improvement, arguing that we should think of their relationship as one that is often entangled and reciprocal, rather than discrete and hierarchical. The next section argues that both the practice of healthcare and deliberative democratic reasoning should be used to help values-improve health systems.

Means-improvement questions are generic to all systems that aim to produce goods or other outputs. Questions of speed, price, accuracy, and resilience need to be considered, whether the system to be improved is a health system, or a restaurant, or a factory production line.^9^ Systems, and also technologies more generally, are typically composed of sub-elements which are themselves technologies or systems, and this encapsulation can be several layers deep. Sub-elements can themselves be means-improved, and this iterates down to sub-sub-processes and beyond (Arthur 2009). While cost-effectiveness analysis can be used to drive means-improvement, it would be a mistake to think that successful means-improvement occurs only where cost-effectiveness analysis is used, whether in health systems or elsewhere.

Indeed, measuring and improving all component elements in a system against the same set of values and the same standards of efficiency (as in cost-effectiveness analysis) is the exception, rather than the rule in means-improvement. It is much more common for the standards used in means-improvement to be local and path-dependent, building from what has been done before and attempting to use this as the basis for improvement—whether it is making glass for a mobile phone screen more scratch resistant, or improving the specificity of a diagnostic assay, or redesigning a staff rota so that it is better able to ensure the right coverage of staff skills while respecting holiday preferences.

One implication is that we should move away from thinking of means-improvement as an unusual activity that can only be undertaken by highly skilled professionals in special circumstances, towards thinking of it as an everyday activity. A variety of different kinds of means-improvement will always already be being pursued simultaneously at different levels of the hierarchical structures of a well-functioning health system. At the highest level, debates may be taking place about how to improve interoperability between different computer systems that store and process patient data; at mid-level, training exercises may be taking place to test a region’s major incident plan; at the most local level a particular team in a hospital may meet to discuss how to change their operating procedures in the light of a revised best practice guideline.

A set of changes to a health system will count a successful means-improvement only if it allows the system as a whole to move in the direction of its values, as articulated and reconciled through processes of values-improvement. Means-improvement thus presupposes an account of values-improvement, and what counts as successful means-improvement will also shift if the values as articulated in a process of values-improvement change. For example, if a values-improvement process leads a health system to place greater emphasis on reducing health disparities between different ethnic groups, then this may require a series of means-improvements such as to the ways in which ethnicity data is coded and recorded, and the ways in which health information is tailored to different communities.

While values-improvement specifies the target for means-improvement at any particular time, it should not be thought of as completely separable from and prior to means-improvement. Means-improvement also shapes values-improvement, both by opening up new possibilities, and by problematising existing values. Sometimes successful means-improvement will provide the opportunity better to specify and reconcile the values that the system is aiming to instantiate and to promote. For example, improvements in anonymisation technologies, and in building Trusted Research Environments make it far easier to do necessary research and planning using linked patient level data in a way that is compatible with strong protection of patient privacy—thus making it significantly easier to reconcile previously conflicting ethical demands of maintaining confidentiality and facilitating improvements to patient care.

Conversely, if a system is unable adequately to deliver the vision specified by a process of values-improvement, even after diligent means-improvement, then this may lead to a requirement for further values-improvement. For example, if an outcome turns out to be much more expensive to produce than initially envisaged, or proves impossible to produce without an unacceptably high rate of errors, or its pursuit undermines other goals of the system, then it is reasonable to assume that this should lead to a rethink of the values-improvement and prioritisation process that led to recommending it in the first place. Perhaps there is a different and more feasible way of pursuing the good in question; or perhaps it may be decided that while the good is correctly deemed important, it is not currently possible to make enough progress for it to be worthwhile as a goal.

Noticing the ways in which economic techniques such as cost-effectiveness analysis are only some of many approaches to means-improvement raises an important question, which I cannot fully address here, about when contextual approaches to means-improvement will be sufficient, and when an approach that allows for easier comparison and ranking should be preferred. Clearly, it will often be the case that what is believed to be best practice locally may no longer seem so good when compared more broadly, and when its systemic effects are mapped; but equally obviously, an approach such as cost effectiveness analysis that makes comparison tractable through homogenisation may struggle to be faithful to some values widely taken to be central to healthcare practice, as the next section explores.^10^

## How the practice of healthcare should shape improvement processes

Human and non-human elements within a system should be treated differently in improvement projects. Machinery is replaceable: it is perfectly reasonable for an office manager to decide to allow a photocopier to be operated in excess of its monthly work cycle, in the knowledge that this will make it more likely to break down, and to require fixing by an engineer. It is very different to ask (or demand) that human beings work beyond a safe workload limit over a prolonged period. Human beings are not replaceable, and a health system cannot and should not price-in the cost of staff moral injury and breakdown. Maximum speed and maximum efficiency thus should not be taken to mean the same thing in the context of the human elements of a system as for the non-human ones. Maximum speed and efficiency for a machine is determined mechanically, but when it comes to the human elements of a system, maximum speed and efficiency must be constrained by and be responsive to the needs, values and moral standing of all those who are stakeholders—whether as patients or as healthcare workers. Failing to consider the role played by the human will have instrumental costs if sick leave and staff turnover increase, and will also be an ethical failing in itself.

Additional core normative expectations arise from healthcare as a social practice. These values, which we can think of as the residue of former attempts at values-improvement, shape the institutions and values into which clinicians are socialised, and shape also societal responses to suffering and death. Healthcare is commonly agreed to be a practice in MacIntyre’s sense, namely a “coherent and complex form of socially established cooperative human activity” through which practitioners aim to achieve standards of excellence, and where the achievement of these standards of excellence partly determines the shape of this activity (MacIntyre 1981, p. 187). These standards of excellence, while not fixed, centrally include the goals of restoration of health, and the relief of suffering.^11^

These normative expectations go deep, and explain why quality improvement—for example reducing surgical complications or better supporting patients with chronic disease to pursue lives they have reason to value—is already at the heart of clinical practice. These normative expectations also profoundly shape acceptable specifications of values-improvement in health systems, and through this acceptable models of means-improvement. They cannot simply be ignored or sidelined in the pursuit of cost-effectiveness; doing so would be likely to have the effect of undermining some of the core values that the health system is supposed to serve.

MacIntyre distinguishes between internal goods, which are defined by the practice itself and are conducive to excellence within it, and external goods which come from outside the practice and are in tension with it. The distinction is helpful for thinking about the relationship between values-improvement and means-improvement, and as with the photocopier example, helps to shape our understanding of which kinds of means-improvement are ethically acceptable given a health system’s values. In so far as institutional arrangements are perceived to require doctors to subordinate the pursuit of goods internal to the practice of medicine to external goods, then good doctors will feel significant tension or moral distress.

Prioritisation of care is not something inimical to the goods internal to the practice of healthcare, and in some form is arguably required by such goods. Clinician time and attention devoted to one patient means less to be given to others, and given the ubiquity of scarcity, such choices must always be made. However, the fact that care must inevitably be limited comes into tension with the responsiveness to human need and suffering that forms the ethical core of healthcare. It is difficult to be a good doctor or nurse without a willingness to put patients’ needs above one’s own. The values at the heart of the social practice thus may make it morally distressing for clinicians to practice in a way that does not allow them to provide good enough care to their patients.

Suppose that a process of means-improvement increases the cost-effectiveness of care on a ward by increasing the number of patients for which each clinician has responsibility. These reforms also make it more difficult for clinicians to establish a personal relationship with patients, and somewhat increase workplace stress. Conscientious clinicians may respond to such pressures unsustainably by attempting to maintain quality of care through creeping increases of unpaid overtime. Such questions challenge health systems to provide a clearer specification of when rationing of care and attention involves allowing external goods to override goods internal to the practice of medicine, and when it is a responsible way of facing up to tensions within values internal to the practice of medicine. To the extent that the resulting cost savings allow improvements of patient care elsewhere, and the net result is an improvement in the overall quality of the care that the health system is able to provide to patients, it will be a matter for public deliberation whether the reforms are in conflict with goods internal to the practice of healthcare, or rather allow for a better specification of them, given the requirement to meet all patients’ needs.

There may not be universal answers to such questions, and deliberative communities may find different equilibria. One thing that will need to be established through such public deliberation is the extent to which the goals of a health system are everyone’s business, and the extent to which health professionals have expertise about these goals that should be deferred to. There are reasons to think that a democracy’s goals must, as a matter of principle, remain open-ended and subject to public debate unless the goals are for specific reasons excluded from reconsideration. If so, open-endedness of goals is a virtue rather than a vice of democratically controlled institutions, and we should expect both means-improvement and values-improvement to be continual and iterative. It may be a sign of imaginative failure rather than argumentative success if it comes to seem that the goals of a health system have all been fully specified and no longer need further debate. As Dewey put it, “All ends and values that are cut off from the ongoing process become arrests, fixations. They strive to fixate what has been gained instead of using it to open the road and point the way to new and better experiences.” (Dewey 2021, pp. 64–5) Of course, even if all democratic ends are up for renegotiation, and the means to such revised ends can all be improved, not everything can or should be called into question at the same time. Values-improvement in healthcare should be thought of, in the first instance, as an argument about how to take forward the values that are already embedded in healthcare as a practice, and have been articulated by previous attempts at values-improvement within the particular health system.

## Conclusion

Regardless of how a healthcare system is financed, it is unlikely that it will be affordable for it to provide all the care that could be potentially beneficial for all citizens. Scarcity is thus a fact of life within health systems. The important question is how policymakers deal with it: will prioritisation be transparent, fair and effective, or will it end up being implicit, haphazard and self-defeating?

While the NHS is often assumed to be almost a paradigm of the transparent, and the procedurally fair when it comes to healthcare resource allocation, I have suggested that this overstates the case. Use of cost-effectiveness analysis is partial rather than complete, and much more attention is given to the question of when to introduce new interventions than to how to stop doing things that are not very cost-effective. The net result is that the current partial approach to cost-effectiveness has not been sufficient to prevent services from becoming congested and waiting times rising inexorably in ways that are harmful for patients.

Reflecting on challenges such as waiting times and system congestion should lead us to shift from the distribution-focused paradigm presupposed in cost-effectiveness analysis and in philosophical discussions of resource allocation, to a flow-centric one. A flow-centric approach requires mapping of systemic interconnections, and examining whether these could be reconfigured to allow the system as a whole to better to achieve the values it aims to instantiate and promote. Rather than thinking of prioritisation as a static process that can be done on the basis of cost-effectiveness analysis alone, we should shift our focus to means-improvement and values-improvement as iterative and often every-day processes by which health systems are improved.

## Footnotes

1 As As Norman Daniels put it, whenever a healthcare system denies treatment to someone on the grounds of cost “we deny benefits to some individuals who can plausibly claim they are owed them in principle; losers as well as winners have plausible claims to have their needs met”. (Daniels 1994, p. 27)

2 Many countries also suffered broader supply chain failures as a result of Covid-19. While these are important, and also had implications for health outcomes, for ease of exposition, in this paper I focus only on health care systems.

3 The distinction between concept and conceptions was introduced by Rawls. On Rawls’s view the concept of justice is that of ‘a proper balance between competing claims’, and this should be distinguished from conceptions of justice, each of which are a ‘a set of related principles for identifying the relevant considerations that determine this balance.’ (Rawls 1999, p. 9).

4 As is familiar from the literature on the social determinants of health, healthcare is only one of a number of determinants of health. The flows that are relevant to health outcomes are much broader than healthcare. Mapping these flows will allow for a range of sometimes counterintuitive interventions, which will allow us to rethink for example the weight that should be given to prevention as opposed to cure. I have written at length about these issues in (Wilson 2021), however for ease of exposition and reasons of length in this article I focus only on flows within healthcare.

5 Waiting times had been increasing significantly even before the Covid-19 pandemic. The diversion of healthcare resources away from non-Covid treatments exacerbated these problems in 2020–21, but performance against indicators continued to decline once the pandemic subsided. The QualityWatch project provides a comprehensive and accessible overview of the relevant data. See Nuffield Trust and Health Foundation (2022b).

6 These high-level considerations, of course, do not by themselves determine whether what should be done is increase available resources for cataract surgery, or to tighten eligibility requirements. Either could be an improvement for a health system, depending on the range of the existing resource commitments and the opportunity costs of scaling up cataract surgery.

7 A report from the Association of Ambulance Chief Executives, published in December 2021 examined the scale of the harms to patients from ambulance handover delays, concluding that each month over 25,000 patients were waiting for more than four times the target handover time of 15 min, and that these delays had contributed to deaths. (Association of Ambulance Chief Executives 2021)

8 The NHS emergency call handlers triage callouts into four categories: Category 1 (Immediately life-threatening event), Category 2 (Emergency), Category 3 (Urgent) and Category 4 (Non-Urgent). There are targets for mean response time (Categories 1 and 2), and for responding to 90% of the cases within the category (Categories 1, 2, 3 and 4). By June 2022 none of these targets were being met—and in many cases there was a wide gulf between targets and system performance. For Category 1 calls, the target is a mean response time of 7 min, but in June 2022 the mean response time was 10 min 33 s. For Category 2 cases, the target is a mean response time of 18 min, and for 90% of cases to be reached within 40 min. The Category 2 mean response time was 51 min, with the 90% centile response time taking 114 min in June 2022. For an overview, see Nuffield Trust and Health Foundation (2022a). The underlying data is published at NHS England (2022).

9 Means-improvement implies a degree of stability in the environment and the system goals that may not always be present. This degree of stability can be assumed in day-to-day public policy, but there are a variety of scenarios such as the outbreak of a pandemic, or responding to a major terrorist incident or natural disaster, where habitual flows are so disrupted or overwhelmed that means-improvement may not be feasible. There are a variety of circumstances in business too where means-improvement takes a back seat—particularly cases of entrepreneurial activity in fast-shifting markets where there are increasing returns. See Arthur (1996).

10 It is salutary to remember that rearranging processes and institutions in the cause of means-improvement may make things worse, as well as better—if only because of the efficiency costs that reorganisation itself brings. Thus, there are reasons to think that those who wish to successfully improve something as complex as a national health system should factor in the costs and inefficiencies that are associated with system change, and satisfy themselves that the changes will be an improvement even given this overhead. One way of doing so is to attend first to cases where things are clearly not working—either according to the standards by which success has been measured, or where standards by which success has been measured are themselves creating problems (perhaps because they create perverse incentives). (Wilson 2009)

11 Some scholars have attempted to use ideas similar to MacIntyre’s to suggest that medicine has an “internal morality”, and that this provides an objective and static basis for the goals of medicine, rather than these being subject to debate and change. See for example, Pellegrino (2001). On such a view, there may be purposes that are excluded by physicians’ integrity, such as participating in state executions (Miller and Brody 1995). Even if this is correct, for the reasons given below, it is not plausible to think that questions of how to allocate scarce healthcare resources will be settled either by the kind of social practice healthcare is, or by the internal morality of medicine. MacIntyre himself emphasises that the goals of practices will change as society changes: “practices never have a goal or goals fixed for all time… the goals themselves are transmuted by the history of the activity.” (MacIntyre 1981, pp. 193–4; see also Ben-Moshe 2019).
